# Safety and tolerability of KarXT (xanomeline–trospium) in a phase 2, randomized, double-blind, placebo-controlled study in patients with schizophrenia

**DOI:** 10.1038/s41537-022-00320-1

**Published:** 2022-12-03

**Authors:** Christoph U. Correll, Angel S. Angelov, Andrew C. Miller, Peter J. Weiden, Stephen K. Brannan

**Affiliations:** 1 Department of Psychiatry Research, The Zucker Hillside Hospital, Glen Oaks, NY USA; 2 Department of Psychiatry and Molecular Medicine, Donald and Barbara Zucker School of Medicine at Hofstra/Northwell, Hempstead, NY USA; 3 Department of Child and Adolescent Psychiatry, Charité Universitätsmedizin Berlin, Berlin, Germany; 4 Karuna Therapeutics, Boston, MA USA

**Keywords:** Schizophrenia, Schizophrenia

## Abstract

KarXT combines xanomeline, an M_1_/M_4_ preferring muscarinic agonist with no direct D_2_ receptor antagonism, with the peripherally restricted anticholinergic trospium. In EMERGENT-1 (NCT03697252), a 5-week, randomized, double-blind, placebo-controlled, phase 2 study in inpatients with schizophrenia, KarXT met the primary efficacy endpoint, numerous secondary endpoints, and was generally well tolerated. Here, we conducted additional post hoc analyses of safety and tolerability data of KarXT from EMERGENT-1 with a particular focus on adverse events (AEs) that may be associated with muscarinic receptor agonism (nausea or vomiting) or antagonism (dry mouth or constipation). A total of 179 patients received at least one dose of either KarXT (*n* = 89) or placebo (*n* = 90) and were included in the analyses. KarXT was associated with a low overall AE burden. The majority of procholinergic and anticholinergic AEs with KarXT were mild, occurred in the first 1−2 weeks of treatment, and were transient with a median duration ranging from 1 day for vomiting to 13 days for dry mouth. No patients in either treatment group discontinued the study due to any procholinergic or anticholinergic AEs. Incidence of somnolence/sedation AEs with KarXT were low and similar to those in the placebo group. KarXT was associated with no significant or clinically relevant changes in body weight, metabolic parameters, or vital signs. KarXT was generally well tolerated with an AE profile consistent with the activity of xanomeline–trospium at muscarinic receptors.

## Introduction

All currently approved antipsychotics for the treatment of schizophrenia have direct affinity for dopamine (DA) D_2_ receptors and block postsynaptic DA receptors^1^. Direct antagonism of DA D_2_ receptors by currently available antipsychotics is associated with characteristic and predictable adverse events (AEs) ranging from movement disorders (e.g., extrapyramidal symptoms and risk of tardive dyskinesia) to behavioral toxicities (e.g., subjective dysphoria and states of lethargy or indifference)^2^. The degree to which individual antipsychotics lead to these or other problems depends on the extent of D_2_ antagonism, the dose used, and individual patient vulnerability^3^. These safety and tolerability problems have evolved as newer, so-called atypical antipsychotics have become available, but the fundamental problem of AEs related to DA receptor antagonism continues to be a major challenge in the treatment of patients with schizophrenia^4–9^. This situation may be changing, with several investigational treatments for schizophrenia currently under development that are devoid of any direct affinity for DA D_2_ receptors^10,11^.

This paper focuses on the types and patterns of AEs observed in a phase 2 clinical trial of muscarinic receptor agonist KarXT (xanomeline–trospium). Although confirmation from phase 3 studies is needed, data from this study show that KarXT may have a different AE profile than other currently available antipsychotics. The antipsychotic activity of KarXT comes from xanomeline, an M_1_/M_4_ preferring muscarinic receptor agonist devoid of any direct DA D_2_ receptor affinity^12–14^. Xanomeline alone (without trospium) demonstrated antipsychotic properties in patients with psychosis associated with dementia^15^ and in patients with schizophrenia^16^. Despite the efficacy signal, clinical development of xanomeline and other direct muscarinic receptor agonists was previously limited by high occurrences of “procholinergic” AEs (e.g., nausea and vomiting, hypotension and syncope) related to their affinity to peripheral muscarinic receptors^15–17^. These AEs were likely related to activity at postganglionic muscarinic receptors located at the efferent end of the parasympathetic autonomic nervous system located in peripheral tissues^18^. As KarXT, xanomeline with trospium included to mitigate these procholinergic AEs may represent a potential new class of treatment for schizophrenia based on central muscarinic receptor agonist activity.

Trospium on its own is approved by the U.S. Food and Drug Administration and widely used for treatment of overactive bladder^19^. The rationale for the trospium component in KarXT is to mitigate xanomeline’s stimulation of peripheral muscarinic receptors without affecting xanomeline’s desired activity on muscarinic receptors located in the central nervous system. Trospium does not cross the blood-brain barrier and avoids the problems associated with centrally active anticholinergics, most notably cognitive problems^20^. When used with xanomeline, trospium is intended to counteract the peripheral muscarinic activity of xanomeline. A phase 1 study (NCT02831231) in healthy volunteers demonstrated that the addition of trospium to xanomeline reduced the incidence of procholinergic side effects versus xanomeline alone^21^. In the EMERGENT-1 trial (NCT03697252)^22^, a 5-week, randomized, double-blind, placebo-controlled, phase 2 study in inpatients with schizophrenia, KarXT was associated with fewer and less severe procholinergic side effects over a 5-week treatment period compared with those observed historically with xanomeline alone^15,16^. Here, we report additional post hoc analyses of data from EMERGENT-1 to provide a closer look at AEs associated with muscarinic receptor activity, which we categorized as procholinergic and anticholinergic AEs. In particular, data on the severity, onset, and duration of AEs related to muscarinic receptor activity are reported^23^. Finally, we report additional details on other AEs commonly associated with antipsychotic medications, including somnolence/sedation, weight gain, and metabolic parameters^2,5,24,25^.

## Results

### Patients

A total of 250 patients were screened and 182 patients who met enrollment criteria were randomized to KarXT (*n* = 90) or placebo (*n* = 92); 179 patients received at least one dose of either KarXT (*n* = 89) or placebo (*n* = 90) and were included in the safety population. Baseline demographics and characteristics for the intention-to-treat population were previously reported^22^ and were found to be similar between treatment groups and generally comparable to other recent US-based inpatient studies in schizophrenia. The median age of patients included in the safety population was 44 years. Demographics and baseline clinical characteristics were similar between treatment groups among patients younger than the median age of 44 years (Table 1). Among those aged ≥44 years, greater percentages of patients in the KarXT group were male (76.1% vs. 61.4%) and white (21.7% vs. 13.6%) relative to the placebo group; other characteristics were comparable between treatment groups.

**Table 1:** Baseline patient demographics and characteristics (intent-to-treat population).

Characteristic	Median Age <44 y		Median Age ≥44 y	
	KarXT (*n* = 43)	Placebo (*n* = 46)	KarXT (*n* = 46)	Placebo (*n* = 44)
Age, years				
Mean ± SD	34.3 ± 5.3	33.4 ± 6.1	52.1 (4.4)	50.7 (3.8)
Median (range)	34.0 (19‒42)	34.0 (20‒42)	52.5 (44‒60)	51.0 (44‒59)
Male sex, *n* (%)	37 (86.0)	39 (84.8)	35 (76.1)	27 (61.4)
Race, *n* (%)				
Black or African American	30 (69.8)	33 (71.7)	36 (78.3)	36 (81.8)
White	11 (25.6)	10 (21.7)	10 (21.7)	6 (13.6)
Other	2 (4.7)	3 (6.5)	0	2 (4.5)
Body mass index, mean ± SD	27.9 ± 5.2	28.2 ± 4.7	28.2 ± 5.0	31.3 ± 5.6
PANSS score, mean ± SD				
Total	99.0 ± 10.8	97.0 ± 8.8	96.3 ± 8.5	96.4 ± 7.7
Positive symptom subscore	26.7 ± 3.3	26.3 ± 3.4	26.2 ± 3.6	26.2 ± 3.1
Negative symptom subscore	23.3 ± 4.8	23.3 ± 4.9	21.9 ± 3.8	22.5 ± 4.2
PANSS Marder factor score, mean ± SD	22.6 ± 5.0	22.8 ± 5.4	21.9 ± 4.6	22.0 ± 4.5
CGI-S score, mean ± SD	5.0 ± 0.6	4.9 ± 0.7	5.0 ± 0.5	5.0 ± 0.5

*CGI-S* Clinical Global Improvement–Severity, *PANSS* Positive and Negative Syndrome Scale, *SD* standard deviation.

### Procholinergic and anticholinergic AEs

As previously reported, the most common AEs occurring in ≥2% of patients in the KarXT group and at a more than two-fold higher incidence than in the placebo group were the procholinergic AEs nausea (16.9% vs. 4.4%) and vomiting (9.0% vs. 4.4%) and anticholinergic AEs constipation (16.9% vs. 3.3%) and dry mouth (9.0% vs. 1.1%)^22^. The majority of procholinergic and anticholinergic AEs were considered mild in severity (Table 2); none were rated as severe (defined as incapacitating or causing an inability to perform normal activities of daily living). No patients in either treatment group discontinued the study due to any procholinergic or anticholinergic AE. The estimated number needed to harm (NNH) for each procholinergic AE and anticholinergic AE (i.e., the number of patients who need to be treated with KarXT rather than placebo for one additional patient to display the AE) is shown in Table 2. For example, nine patients would have to be treated with KarXT rather than placebo for one additional KarXT-treated patient to display nausea.

**Table 2:** Severity and NNH estimates of procholinergic and anticholinergic AEs (reported by ≥2% of patients in the KarXT group and at >2-fold higher incidence than in the placebo group).

*n*/*N* (%)	KarXT (*n* = 89)			Placebo (*n* = 90)			NNH (95% CI)
	Mild	Moderate	Severe	Mild	Moderate	Severe	
Procholinergic AEs							
Nausea	13/15 (86.7)	2/15 (13.3)	0/15 (0)	3/4 (75.0)	1/4 (25.0)	0/4 (0)	9 (5, 29)
Vomiting	5/8 (62.5)	3/8 (37.5)	0/8 (0)	3/4 (75.0)	1/4 (25.0)	0/4 (0)	23 (9, −36)
Anticholinergic AEs							
Constipation	12/16 (75.0)	4/16 (25.0)	0/16 (0)	2/3 (66.7)	1/3 (33.3)	0/3 (0)	8 (5, 21)
Dry mouth	6/8 (75.0)	2/8 (25.0)	0/8 (0)	1/1 (100)	0/1 (0)	0/1 (0)	13 (8, 65)

*AE* adverse event, *CI* confidence interval, *NNH* number needed to harm.

**Fig. 1:**
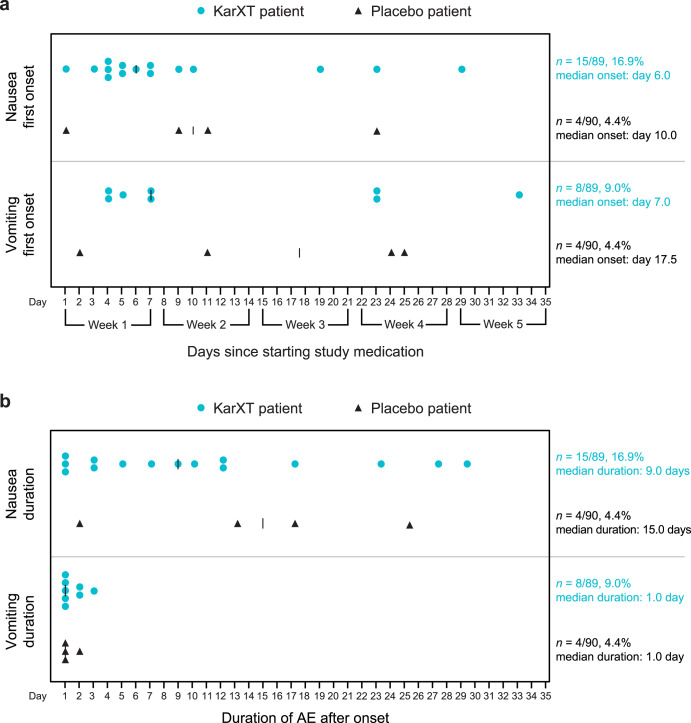
Summary of procholinergic AEs. **a** Time to onset and **b** duration of procholinergic AEs. AE adverse event.

**Fig. 2:**
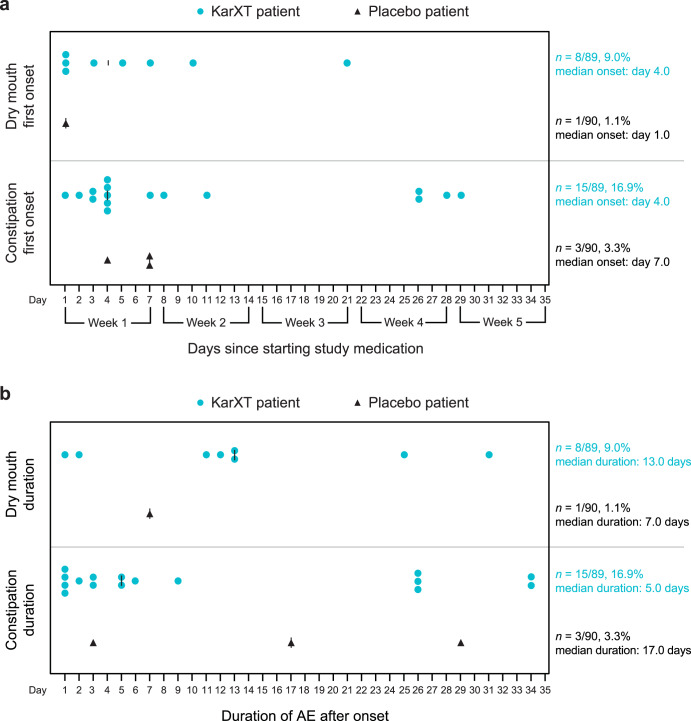
Summary of anticholinergic AEs. **a** Time to onset and **b** duration of peripheral anticholinergic AEs. Duration was calculated as (AE end date−AE start date) +1, except for two instances of constipation that were ongoing at the end of the trial, in which case duration was imputed using end of study date instead of AE end date. AE adverse event.

The time to onset from first dose and duration of procholinergic and anticholinergic AEs are shown in Figs. 1 and 2, respectively. Most of the procholinergic and anticholinergic AEs with KarXT were reported within the first 1−2 weeks of treatment. All procholinergic AEs in the KarXT group were transient, especially vomiting, which resolved within 1 day in most cases and had a maximum duration of 3 days. The median duration of nausea was 9 days with KarXT and 15 days with placebo. Anticholinergic AEs with KarXT lasted a median duration of 13 days for dry mouth and 5 days for constipation compared with 7 days and 17 days, respectively, with placebo. Given the early onset and transient nature of AEs reported in this trial, cholinergic AE rates were similar between the KarXT and placebo groups after the first 3 weeks of the trial.

### Somnolence/sedation AEs

**Fig. 3:**
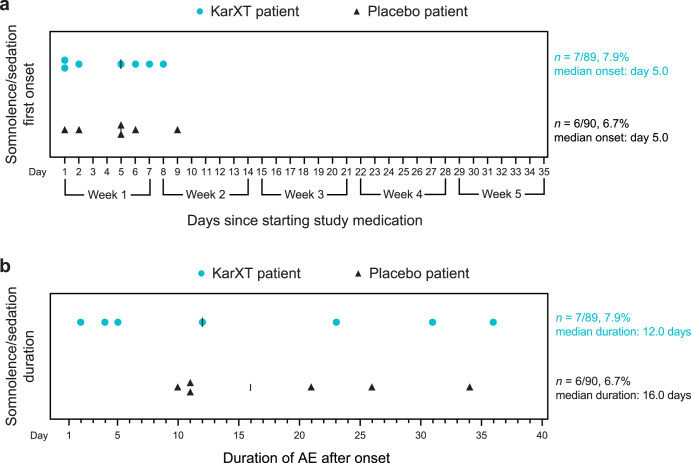
Summary of somnolence/sedation AEs. **a** Time to onset and **b** duration of somnolence/sedation AEs^*^. ^*^There was no overlap in any patients experiencing somnolence or sedation AEs. Duration was calculated as (AE end date−AE start date) +1, except for one instance that was ongoing at the end of the trial in which case duration was imputed using the participant’s end of study date instead of AE end date. AE adverse event.

The incidence of somnolence/sedation AEs was 7.9% in the KarXT group (*n* = 7/89) and 6.7% in the placebo group (*n* = 6/90). The severity of somnolence/sedation AEs was rated as mild in 3/7 patients (42.9%) and moderate in 4/7 patients (57.1%) in the KarXT group compared with mild in 5/6 patients (83.3%) and moderate in 1/6 patients (16.7%) in the placebo group. The median time to first onset of somnolence/sedation AEs was the same between KarXT (5.0 days) and placebo (5.0 days) groups, with all events being reported within the first 9 days of treatment (Fig. 3a). All somnolence/sedation AEs were transient and resolved during the course of the study with a median duration of 12 days in the KarXT group and 16 days in the placebo group (Fig. 3b).

### Vital signs

**Fig. 4:**
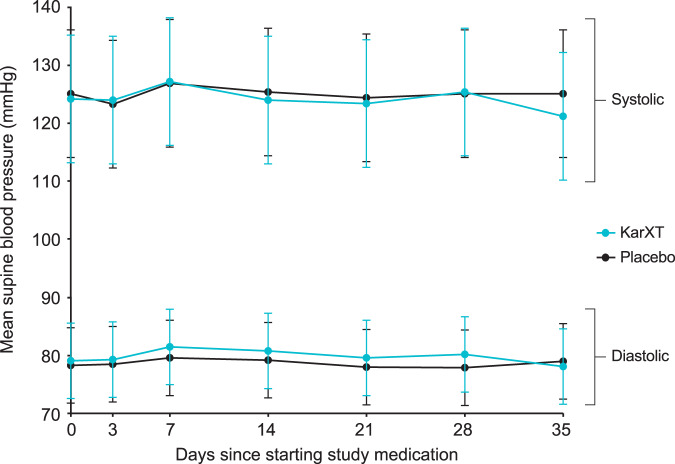
Observed change in supine systolic and diastolic blood pressure (± SD). On day 0, blood pressure was measured within 2 hours of the first dose of study medication. During the treatment period, blood pressure was measured 2 (± 1) hours after morning dose of study treatment, except on day 35 when measurements were taken in the morning approximately 12 hours after the last dose of study medication on day 34. SD standard deviation.

**Fig. 5:**
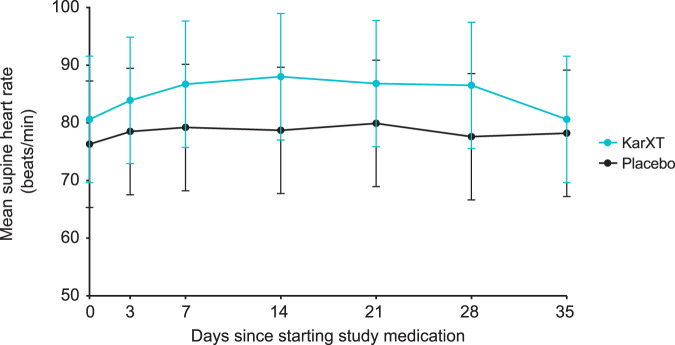
Observed change in supine heart rate (± SD). On day 0, heart rate was measured within 2 hours of the first dose of study medication. During the treatment period, heart rate was measured 2 (± 1) hours after the morning dose of study treatment, except on day 35 when measurements were taken in the morning approximately 12 hours after the last dose of study medication on day 34. SD standard deviation.

There were no significant or clinically meaningful changes between day 0 (treatment initiation) and day 35 in any of the five vital signs, including mean supine systolic blood pressure, diastolic blood pressure, or heart rate in either the KarXT or placebo groups (Figs. 4 and 5). Mean change from day 0 to day 35 in supine systolic blood pressures was −3.9 mmHg in the KarXT group versus −0.2 mmHg in the placebo group (Fig. 4). Mean change from day 0 to day 35 in supine diastolic blood pressures was −1.4 mmHg in the KarXT group versus 0.5 mmHg in the placebo group (Fig. 4). Similarly, no significant or clinically meaningful changes between day 0 and day 35 were seen for orthostatic systolic or diastolic blood pressure or heart rate. For supine heart rate, mean change from day 0 to day 35 was 0.4 beats/min in the KarXT group versus 2.8 beats/min in the placebo group (Fig. 5). While mean changes were not statistically significant or clinically meaningful, KarXT was associated with a trend of increased heart rate that peaked at day 14 and decreased in magnitude for the remainder of the trial.

### Weight gain

A total of two (2.2%) patients in the KarXT group and five (5.6%) patients in the placebo group experienced a > 7% change in body weight. As previously reported, in the overall safety population, the mean change (± SD) from baseline in body weight at week 5 was 1.5 ± 2.8 kg in the KarXT group and 1.1 ± 3.5 kg in the placebo group^22^. The mean change (± SD) in body weight from baseline to week 5 was similar between KarXT and placebo groups among patients aged <44 years (1.2 ± 2.8 kg vs. 0.3 ± 3.8 kg, respectively) and among those aged ≥44 years (1.7 ± 2.9 kg vs. 1.9 ± 3.1 kg, respectively). Analysis of data by sex revealed comparable small changes in body weight across KarXT and placebo groups in both male (1.4 ± 3.0 kg vs. 1.0 ± 3.8 kg, respectively) and female (1.6 ± 2.3 kg vs. 1.4 ± 2.8 kg, respectively) patients.

### Metabolic parameters

There were no significant or clinically meaningful changes from baseline to week 5 in cholesterol, blood glucose, or triglyceride concentrations (Table 3). Total mean nonfasting cholesterol levels for both KarXT (178.7 mg/dL) and placebo (183.6 mg/dL) remained within the normal reference range (<200 mg/dL) at study end. Similarly, mean serum triglyceride concentrations for both KarXT (120.3 mg/dL) and placebo groups (123.8 mg/dL) remained within the normal reference range (<150 mg/dL) at the study end. Mean blood glucose levels at the study end were 103.4 mg/dL in the KarXT group and 101.9 mg/dL in the placebo group; no patients in either arm had an AE of increased blood glucose level that was considered related to treatment.

**Table 3:** Mean change from baseline to week 5 in metabolic parameters.

	KarXT (*n* = 89)	Placebo (*n* = 90)
Total Cholesterol, mg/dL	0.8 ± 27.71	−4.9 ± 27.04
Glucose, mg/dL	9.8 ± 32.23	12.5 ± 41.56
Triglycerides, mg/dL	13.4 ± 51.88	−5.7 ± 75.86

Plus-minus values are mean ± standard deviation.

### AE incidence by age

Additional analyses of AEs were performed. The main finding was the overall incidence of AEs with KarXT did not differ by age (53.5% for patients age <44 years vs. 54.3% for patients age ≥44 years; Table 4). In contrast, in the placebo group, the incidence of AEs was substantially lower in older patients (age ≥44 years; 34.1%) than in younger patients (age <44 years; 52.2%). The distribution of AE severity did not appear to show any differences between groups.

**Table 4:** Incidence and severity of treatment-emergent AEs by median age.

*n* (%)	Median Split Age <44 y		Median Split Age ≥44 y	
	KarXT (*n* = 43)	Placebo (*n* = 46)	KarXT (*n* = 46)	Placebo (*n* = 44)
Any AE	23 (53.5)	24 (52.2)	25 (54.3)	15 (34.1)
Any mild AE	22 (51.2)	19 (41.3)	19 (41.3)	13 (29.5)
Any moderate AE	6 (14.0)	10 (21.7)	9 (19.6)	3 (6.8)
Any severe AE^a^	1 (2.3)	1 (2.2)	0	0

^a^A severe AE was defined as any event that was incapacitating or caused an inability to perform normal activities of daily living.

*AE* adverse event.

## Discussion

The additional analyses from the phase 2 EMERGENT-1 study presented here underscore the overall favorable safety and tolerability profile of KarXT in patients with acute symptoms of schizophrenia treated in the inpatient setting. The safety and tolerability findings in this study were consistent with both pro- and anti-muscarinic receptor activity. Although the safety and tolerability profile of xanomeline has been previously reported, this is the first clinical trial of xanomeline using the KarXT formulation in patients with schizophrenia. These results support the findings from an initial phase 1 trial that demonstrated that the addition of trospium to xanomeline mitigates procholinergic AEs. Although KarXT was associated with some procholinergic AEs, they appeared to be more limited in incidence, severity, and duration than in prior studies using xanomeline alone^15,16^. The presumed benefits of trospium were tempered by the presence of anticholinergic AEs represented by dry mouth and constipation. Presumably those AEs were related to trospium since they are consistent with the known AEs associated with use of trospium in overactive bladder^19^. Although balancing of procholinergic versus additional anticholinergic AEs remains an important question for the phase 3 studies, these results are reassuring given that both the cholinergic and anticholinergic AEs were all mild or moderate in severity, were often of short duration, and did not lead to any discontinuations from the trial. Furthermore, anticholinergic AEs are well known from use of anticholinergics for extrapyramidal symptoms, but without the cognitive problems associated with the central anticholinergic effects of anti-Parkinsonian anticholinergic medications^26,27^. Although KarXT has not been studied in head-to-head trials with other antipsychotics and caution is warranted when comparing trials, these results suggest that KarXT is well tolerated and its profile may differ substantially from other antipsychotics commonly prescribed for schizophrenia^2,5^, as reflected in the lack of either substantial incidences of or clinically meaningful somnolence/sedation, weight gain, or changes in metabolic parameters, during the 5-week treatment period.

Despite evidence of antipsychotic efficacy in patients with psychosis associated with dementia^15^ as well as schizophrenia^16^, the earlier clinical development of xanomeline was limited by the incidence and severity of procholinergic AEs, in particular gastrointestinal effects, excessive sweating, and hypersalivation^18^. EMERGENT-1 provides clinical evidence in patients with schizophrenia that combining xanomeline with the peripherally acting anticholinergic trospium can reduce the risk of these AEs in terms of incidence, severity, and clinical relevance compared with the risk with xanomeline alone in prior trials^22^. Although procholinergic and anticholinergic AEs were more frequent in the KarXT group than in the placebo group, the majority of these events were mild in severity, none were severe, and none led to study discontinuation. Here we report that the majority of these AEs appeared within the first week of treatment and lasted a median duration ranging from 1 day for vomiting to 13 days for dry mouth. In fact, all of these AEs occurred in the placebo arm as well. Although these AEs were more common in patients treated with KarXT than placebo, the severity of these AEs was not very different between the two treatment groups and the median duration of nausea and constipation was somewhat longer in the placebo arm than in the KarXT arm. Given the early onset and transient nature of AEs reported in this trial, cholinergic AE rates were similar between the KarXT and placebo groups after the first 3 weeks of this trial. The finding that some AEs were more frequent (eg, ≥7% body weight increase) or lasted somewhat longer (eg, nausea, constipation) in the placebo arm is interesting. However, differences were generally small and due to the small number of patients in this phase 2 study, and further data from the pending phase 3 studies are needed to evaluate these tolerability signals in more detail. Additionally, the ongoing open-label, long-term safety study will provide further information regarding time trajectories of AEs with KarXT.

Somnolence and sedation can lead to substantial additional morbidity for patients with schizophrenia^9^, and some currently available antipsychotics can exacerbate these symptoms^28^. In EMERGENT-1, rates of somnolence/sedation AEs with KarXT were low and similar to those in the placebo group. All patients with somnolence/sedation AEs experienced onset within the first 9 days of treatment. These AEs were typically transient and all events resolved during the course of the study. These results suggest that, unlike antipsychotics with potent central antihistaminergic properties^2,9^, the central muscarinic receptor effects of KarXT might not be as disruptive to patients’ daily activity and sleep-wake cycle, a finding that requires further evaluation in ongoing short- and long-term studies.

Muscarinic receptors are present on vascular smooth muscle cells and known to influence cardiovascular activity^23^, and other muscarinic drugs in development have been reported to have effects on blood pressure and heart rate^29^. Our analyses show that KarXT was associated with no significant or clinically meaningful changes between baseline and week 5 in mean supine systolic blood pressure, diastolic blood pressure, or heart rate in either the KarXT or placebo groups. All three measures were slightly lower at week 5 compared with baseline in both treatment groups; these results are consistent with findings from a phase 1 study with KarXT^30^. Unlike the other cardiovascular parameters, there was an initial increase in heart rate relative to placebo that was observed during the first weeks of KarXT treatment that then diminished relative to placebo by the end of the 5-week study. The slight drop in observed blood pressure and heart rate with KarXT at week 5 may be because the final measures were approximately 12 hours after the last dose of study medication, whereas earlier measures were approximately 2 hours after dosing.

Metabolic disturbances, weight gain, and cardiovascular disease are common in patients with schizophrenia and are main contributors to increased morbidity, higher mortality rates, and shorter life expectancy;^31–37^ for example, more than one-third of patients with chronic schizophrenia who are treated with antipsychotics experience metabolic syndrome, an important risk factor for cardiovascular and metabolic disorders^38,39^. Many antipsychotics commonly prescribed for schizophrenia, especially second-generation antipsychotics, are well known to have adverse effects on body weight, glucose, or lipids^25,40^, even over the course of the typical acute timeframe of short-term pivotal clinical trials. In contrast, the overall pattern in this study is consistent with KarXT having little or no effect on weight, total cholesterol, glucose, or triglycerides compared with placebo during 5 weeks of medication exposure. Similar small increases in weight (all < 2 kg) were observed in KarXT and placebo groups among patients aged <44 years and in those aged ≥44 years as well as in both male and female patients. In addition, the percentage of patients who gained >7% of their body weight was lower in the KarXT versus placebo groups. These results suggest that there were no notable propensities toward weight gain with KarXT dependent on age or sex during the course of this short-term, 5-week study.

Finally, the overall incidence of treatment-emergent AEs (TEAEs) for KarXT patients did not appear to be related to age, with an overall AE incidence of 53.5% for patients age <44 years and 54.3% for patients age ≥44 years. The same split by median age in the placebo group showed an incidence of 52.2% for patients age <44 years and 34.1 for patients age ≥44 years. These analyses by age were meant as preliminary, and the lower AE incidence among older patients in the placebo group was unexpected. Given the difficulty in interpreting these findings and relative lack of other clinically concerning AEs, no further analyses were performed, given the fairly small number of patients. This finding warrants further exploration in analyses of data from the phase 3 trials.

In summary, EMERGENT-1 demonstrated that KarXT had a favorable safety and tolerability profile over a 5-week treatment period with a low risk for many AEs commonly seen with currently available antipsychotics in patients with schizophrenia. As the first registrational study, these results are encouraging and highlight the potential for KarXT and a new class of treatments for schizophrenia, the muscarinic receptor agonists. Although the results of this study are limited by the short-term nature of the study, most of the AEs reported here typically become evident in clinical trials of similar duration, and the median duration of these AEs before tolerance developed and the AEs were not reported anymore was generally relatively short. The potential introduction of muscarinic receptor agonists, such as KarXT, to patients with schizophrenia and other neuropsychiatric disorders may shift the safety and tolerability profile from AEs commonly associated with postsynaptic dopaminergic antagonist and partial agonist treatments (e.g., dystonia, Parkinsonism, akathisia, tardive dyskinesia) to AEs related to pharmacodynamic effects related to peripheral or central muscarinic receptors. Results from the phase 3 EMERGENT-2 (NCT04659161) and EMERGENT-3 (NCT04738123) studies, as well as longer-term follow-up from the EMERGENT-4 (NCT04659174) and EMERGENT-5 studies (NCT04820309), are needed to confirm and extend these findings and further characterize the safety and tolerability profile of KarXT during longer exposure.

## Methods

### Study design

EMERGENT-1 (NCT03697252) was a randomized, double-blind, placebo-controlled phase 2 study of KarXT in adult inpatients with schizophrenia. The study methods, population, safety, and prespecified primary and secondary endpoints have been previously published^22^. Briefly, after a 7-day screening period, patients who met entry criteria were randomized 1:1 to receive either oral KarXT or matched placebo twice daily for 5 weeks of treatment in an inpatient setting. The dosing schedule of KarXT (mg xanomeline/mg trospium) was flexible, starting with 50 mg/20 mg twice daily and increasing to a maximum of 125 mg/30 mg twice daily. The study was conducted in the United States from September 2018 to September 2019.

The study was conducted in accordance with the International Conference on Harmonization Good Clinical Practice Consolidated Guidelines, the Declaration of Helsinki guidelines, and applicable regulatory requirements. The study protocol, including the informed consent form, and amendments were approved by a central institutional review board (Copernicus Group, Cary, NC, USA). All patients provided written informed consent prior to participation. The study protocol is available at: www.nejm.org/doi/suppl/10.1056/NEJMoa2017015/suppl_file/nejmoa2017015_protocol.pdf.

### Patients

EMERGENT-1 enrolled 182 adult patients aged 18−60 years who had a primary diagnosis of schizophrenia based on the *Diagnostic and Statistical Manual of Mental Disorders*, fifth edition^41^. Key inclusion criteria included recent worsening of positive symptoms that warranted hospitalization, a Positive and Negative Syndrome Scale (PANSS)^42^ total score ≥80, and a Clinical Global Impression–Severity Scale^43^ score of ≥4 (moderately ill). Patients were excluded if they had a primary disorder other than schizophrenia within the 12 months prior to screening, a history of treatment resistance to antipsychotic medications, or a decrease in PANSS total score ≥20% between screening and baseline.

### Safety assessments

Safety assessments were assessed through AE monitoring and measuring weight, nonfasting clinical laboratory values, and vital signs. AEs that occurred during the treatment period were defined as those starting or worsening after the first dose of KarXT or placebo to the end of study. Standard safety reporting data are found in the primary publication^22^. The current analyses in this report provide additional qualitative information. Analysis was conducted specifically on “procholinergic” and “anticholinergic” AE categories, which were defined as AEs that are clearly associated with muscarinic receptor agonism (nausea or vomiting) or antagonism (dry mouth or constipation) were selected. Blood pressure and heart rate were measured on the day of treatment initiation (day 0), day 3, day 7, and weekly thereafter; during the treatment period, vital sign measurements were taken 2 (± 1) hours after the morning dose of study treatment, except on day 35 when measurements were taken in the morning approximately 12 hours after the last dose of study medication on day 34.

### Statistical methods

All safety and tolerability analyses were performed in the safety population, defined as all patients who received at least one dose of study drug. AEs that emerged during the study were rated for severity according to the treatment group and time point. Analyses employed descriptive statistics. The incidence and severity of TEAEs were analyzed separately for patients below and at or above the median age for each treatment group. For change from baseline to last visit calculations, the baseline was defined as the last measurement prior to the first dose of drug received. The AE duration was calculated as AE end date-AE start date +1, except for two instances of constipation and one instance of somnolence/sedation that were ongoing at the end of the trial, in which case duration was imputed using each patient’s end-of-study date instead of AE end date. The NNH estimates were calculated as 1/(incidence rate on KarXT-incidence rate on placebo), then rounded up to the nearest integer value. Weight gain from baseline to week 5 was described separately for patients below and at or above the median age for each treatment group and for male and female patients.

### Reporting summary

Further information on research design is available in the Nature Research Reporting Summary linked to this article.

## Data Availability

Data meeting the regulatory statutes is available at clinicaltrials.gov.
